# Mechanistic modelling of viral spreading on empirical social network and popularity prediction

**DOI:** 10.1038/s41598-018-31346-0

**Published:** 2018-09-03

**Authors:** Sijuan Ma, Ling Feng, Choy-Heng Lai

**Affiliations:** 1 Department of Physics, National University of Singapore, Singapore, 117551 Singapore; 2 Computing Science Department, Institute of High Performance Computing, A*STAR, Singapore, 138632 Singapore; 3 Centre for Quantum Technologies, National University of Singapore, Singapore, 117543 Singapore

## Abstract

Online social networks are becoming major platforms for people to exchange opinions and information. While spreading models have been used to study the dynamics of spreading on social networks, the actual spreading mechanism on social networks may be different from these previous models due to users’ limited attention and heterogeneous interests. The tractability of the spreading process in social networks allows us to develop a detailed and realistic model accounting for these factors. In addition, the empirical social networks have higher order correlations among node degrees, especially for directed networks like Twitter, that could affect the dynamics of spreading. Based on the analysis of the retweet process in the empirical Twitter network, we find both non-trivial correlations in network structures and non-standard spreading mechanisms for viral tweets. In particular, there is a strong evidence of information overload for retweeting behaviors that is not in line with the standard spreading model like the SIR (Susceptible, Infectious and Recovered) model, and can be described by a sublinear function. From these empirical findings, we introduce an intrinsic variable “attractiveness” to the message, describing the overall propensity for any node to retweet the message, and present the analytical equations to solve such an empirical process, validated through numerical simulations. The results from our model is consistent with findings from the empirical Twitter data. Our analysis also indicates a close relationship between the spreading sub-network structure and the final popularity of the information, leading to a method to predict the popularity of tweets more accurately than existing models.

## Introduction

With the development in communication technologies, online social networks like Facebook and Twitter are essential platforms for information spreading and opinion exchange. Studies of information diffusion suggest that interplay between human cognitive limits and network structure differentiates the spread of information from other social contagions^1,2^. Considering the considerable potential for social media marketing, plenty of interest is now being focused on the spreading mechanism in social networks^3–6^. Some involve analyzing large amounts of empirical data^7–11^, and others formulate predictions of the popularity of a particular piece of information^12–14^.

Reference^15^ provides a generative model for online sharing behaviour and distinguishes two distinct factors affecting meme popularity: the memory time of users (the visible time period of messages retweeted by the user) and the connectivity structure of the social network. On the other hand, inspired by epidemic spreading, the Susceptible-Infected-Recovered (SIR) model^16^ is frequently used to represent the spread of information^17,18^. Although the spreading processes of disease, opinion, and information all share some similarities, fundamental differences remain. In epidemic spreading^19–21^, every susceptible individual coming in contact with an infected individual has the same probability of being infected and the spreading of one specific disease is usually assumed to be independent of other diseases. In contrast, the abundance of information flowing through online social network has made the information ecosystem highly competitive^15,22,23^. In fact, the users’ behavioural activity is highly heterogeneous and superfluous messages compete for attention in online social systems. Although several studies described the dynamics of information flow in popular communication media^8,10,24^, straightforward theoretical framework that addresses the users’ heterogeneous behaviour and attention competition is still absent.

On the other hand, as one of the most interesting problems in modeling information spreading, popularity prediction have received a fair amount of attention in recent years. Lerman *et al*.^25^ modeled the user voting process on Digg by considering the visibility of the online content. Hong *et al*.^26^ formulated the popularity prediction as a classification problem. Some studies used temporal and structural features to predict popularity in microblogging networks^27–29^. However, a comprehensive and simplified method based on a mechanistic model is still absent.

In this paper, we focus on modeling the “viral” spreading of messages in the supercritical phase by imposing a constraint on the messages we use in the study, such that the spreading reaches much further beyond the nearest neighbours of the seed node. The total retweet number of each message is at least 50% of the number of followers of the seed node of the message. Analysis of the final retweet trees shows that most of the messages have diameters larger than 20, signifying a supercritical phase of spreading reaching much further from the seed node’s neighbours. Based on the empirical data of the Twitter network crawled by Twitter API^30^, we extract the detailed network structure and behavioral characteristics of retweeting users. We formulate the retweet probability of an incoming message based on the combination of retweet activity and attention competition. In addition, we introduce the factor (variable) “attractiveness” to describe the intrinsic quality of a message, that arouses the user’s interest. Subsequently, we propose a mechanistic model involving the heterogeneous retweet probability based on mean field theory. The mean field theory assumes the influence acting on different individual components is the average effect in the system, ignoring certain individualities of each component to simplify the analysis^31^. The model can be solved by a self-consistent probability method to determine when the information will spread out. Intuitively, information will spread out when the giant out-component size is larger than zero. It turns out that our model is reasonable and agrees with empirical spreading as compared with the original SIR model. Furthermore, we compare the characteristic properties deduced from our model and the SIR model with the empirical analysis of Twitter messages, and the results show that our model better matches the empirical spreading.

In order to investigate the information flow, we derive the retweet tree of each message from empirical data. An interesting finding shows that the retweet popularity (final number of retweets) is strongly correlated with the statistical characteristics of the retweet tree. We use numerical iteration method to analyze the branching process based on our mechanistic model. The results show the same pattern between the retweet popularity and the kurtosis of the out-degree distribution of the retweet tree. Inspired by this, we propose an effective method to predict the popularity using the exact out-degree distribution of the retweet tree in the early stage of spreading. Our method gives higher prediction accuracy compared to other existing methods.

## Mechanistic Modelling from Empirical Analysis of Twitter Network

### Empirical analysis on Twitter structure and user behaviors

#### Twitter network structure

**Figure 1:**
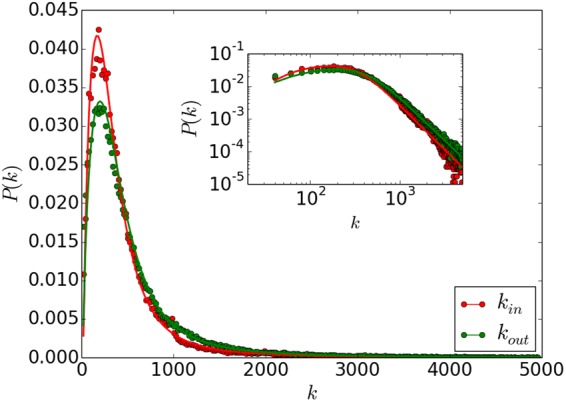
The in-degree and out-degree distributions of the Twitter network. The points represent the discrete probabilities falling into corresponding bins from empirical analysis with a bin size of 20. The solid line represents the fitted formula.

In order to explore the Twitter network structure, we have crawled the followship and friendship of about one million accounts. The Twitter network is a large directed network with a high average degree. The in-degree *k*_*in*_ of a node is the number of friends and the out-degree *k*_*out*_ represents the number of followers. As shown in Fig. 1, the *k*_*in*_ and *k*_*out*_ distributions can be fitted in the following form with different fitting parameters. The value of (*a*, *b*, *c*, *d*) equals (0.08, 300, −12, 3.0) for *k*_*in*_ and (0.062, 360, −8, 3.0) for *k*_*out*_:

1 $$P({k}_{in})=a\times \,\tanh \,{(\frac{{k}_{in}+c}{b})}^{d}\times {\frac{{k}_{in}}{b}}^{-2.7},$$

2 $$P({k}_{out})=a\times \,\tanh \,{(\frac{{k}_{out}+c}{b})}^{d}\times {\frac{{k}_{out}}{b}}^{-2.6}.$$

The peaks in Fig. 1 indicate that the numbers of friends and followers tend to concentrate in the interval of [200, 300]. As for the large degree values, the degree distribution approaches a power law^32^.

**Figure 2:**
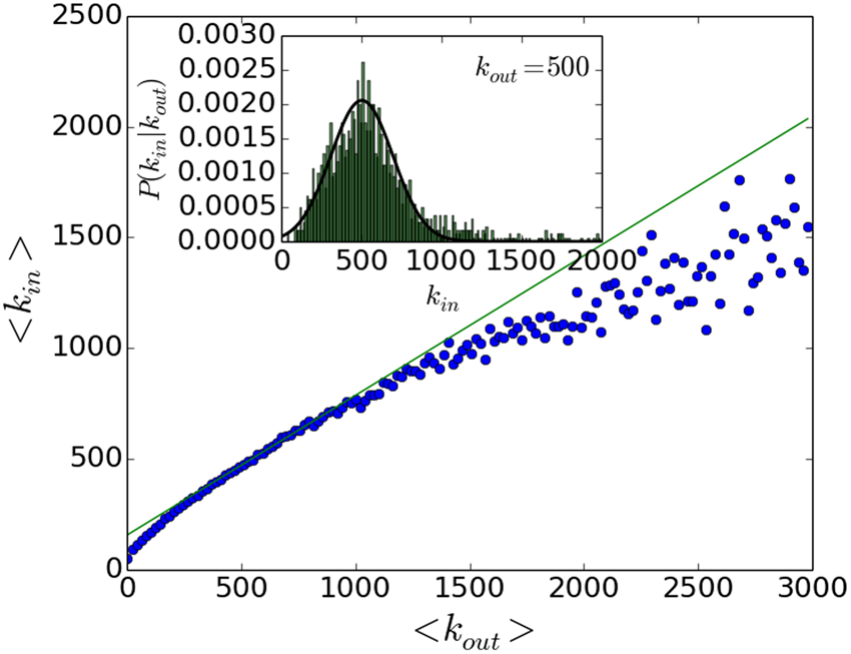
The in-degree and out-degree correlation of the nodes in the Twitter network. Each point is the average value of the in-degree for nodes with out-degrees in a binned interval (horizontal axis). The bin size is 20. The figure shows that the average value of the in-degree is approximately linearly related to the average value of the out-degree. The inset shows the *k*_*in*_ distribution for nodes with *k*_*out*_ ≈ 500, and it is fitted to the normal distribution.

In addition, we find that there is a positive correlation between the in-degree and the out-degree. As shown in Fig. 2, 〈*k*_*in*_〉 is approximately linearly related to 〈*k*_*out*_〉. The average of *k*_*in*_ is calculated for users that have out degrees falling into the corresponding bins. The inset of Fig. 2 shows the *k*_*in*_ distribution for nodes with *k*_*out*_ ≈ 500, and it is fitted to the Normal distribution (solid line) as

3 $$P({k}_{in}|{k}_{out})\sim {\mathscr{N}}(\mu ={k}_{out}\times 0.62+147,\,{\sigma }^{2}=\frac{{k}_{out}}{2}).$$

We find that the degree correlation between neighbors is weak, so we assume that the assortativity is zero in the whole network.

#### Behavioural activity of Twitter users

**Figure 3:**
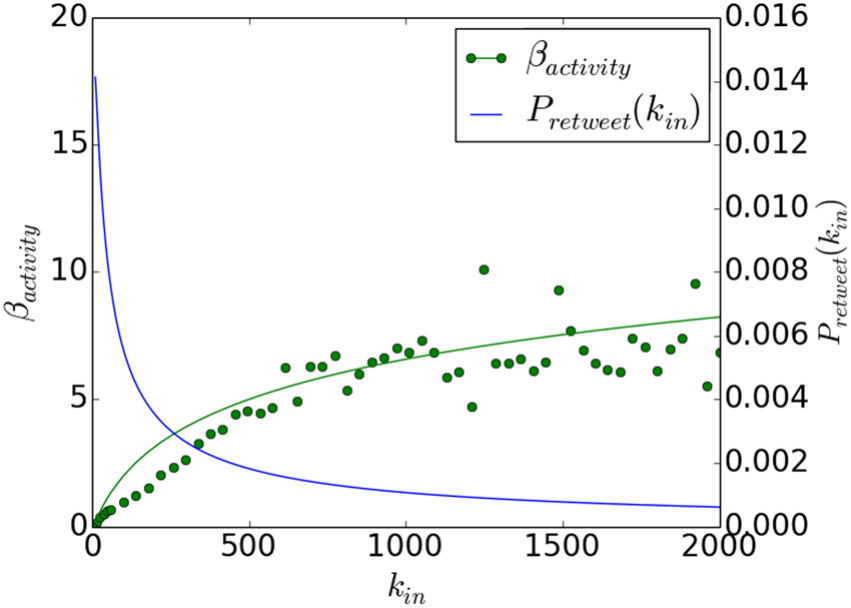
The behavioural activity and retweet probability as a function of in-degree. Green dots represent daily retweet frequencies from the empirical Twitter data, fitted by the green line. The blue line shows the retweet probability as a function of the user’s in-degree.

Behavioural activity is a predominant factor that has influence on the retweet probability. We use the daily retweet frequency as a measurement of the retweet activity and investigate its dependence on the in-degree. In Fig. 3, the green line shows that a user with more friends has higher retweet activity and the correlation can be fitted with the following sublinear formula:

4 $${\beta }_{activity}({k}_{in})=a\times \,\mathrm{ln}\,(\frac{{k}_{in}}{d}+b)+c.$$

Consequently, the daily volume of incoming information can be represented by 〈*β*_*activity*_〉 × *k*_*in*_ under the condition that the assortativity approximates to zero.

It has been suggested that users’ interests on topics affect their behavior in social media^33,34^. To describe the information intrinsic quality that contributes to a user’s interest, we introduce the variable “attractiveness” *A*. The retweet probability of a particular tweet *j* by a node with in-degree *k*_*in*_ is then determined by the daily retweet frequency, the total volume of incoming messages and the intrinsic attractiveness of the message. It can be formulated as follows:

5 $${P}_{retweet}^{j}({k}_{in})=\frac{(a\times \,\mathrm{ln}\,(\frac{{k}_{in}}{d}+b)+c)\times {A}_{j}}{\langle {\beta }_{activity}\rangle \times {k}_{in}}$$

Since the average value of *A* equals 1, such that *A*_*j*_ ≪ *k*_*in*_, the probability is smaller than 1. The probability in Eq. 5 is the retweet probability we used in the discrete model. The denominator in the formula contains the effect of competition. Since the Twitter network structure is fixed and the retweet behaviour is an intrinsic characteristic to each user, and is inferred from empirical data from the average number of retweets for the user, attractiveness is the tunable parameter that changes the retweet probability in our model. For a node with in-degree *k*_*in*_, the sum of retweet probability over all incoming tweets in one day should equals to the daily retweet activity (number of daily retweets for the user):

$$\begin{array}{rcl}\sum _{j=1}^{m}\,{P}_{retweet}^{j} & = & {\beta }_{activity}({k}_{in}),\\ \sum _{j=1}^{m}\,{A}_{j} & = & \langle {\beta }_{activity}\rangle \times {k}_{in}.\end{array}$$

Since the number *m* of incoming retweet in one day is equal to 〈*β*_*activity*_〉 × *k*_*in*_, we have

$$\langle A\rangle =\frac{\sum _{j=1}^{m}\,{A}_{j}}{m}=1.$$

Hence for a specific message, *A* = 1 represents the average attractive level. Intuitively, *A* > 1 implies greater attractiveness, and *A* < 1 corresponds to lower attractiveness.

### Self-consistent probability method for the branching process

The propagation of retweets among users forms a graph. We measure the popularity of a tweet by the size of the largest component in the graph, which is called the giant component in the bond percolation process^35,36^. To investigate how the giant out-component size depends on the retweet probability, we employ the self-consistent probability method for our analytical and numerical analyses, and incorporate our empirical findings into the method.

The spreading of a message is a branching process that starts with the creation by an initial node, followed by its followers retweeting it, and then the followers’ followers, with the process continuing until no more user retweets it. The definition of popularity here is defined as the final number of people retweeting the message in the whole Twitter network. The process is similar to the SIR model in epidemic spreading over a human contact network^37,38^. The three states here have the following interpretations:

- “S”: the Susceptible state. In Twitter it refers to the users that have not retweeted the message.
- “I”: the Infected state. In Twitter it refers to the users that have retweeted the message, and their retweets are still visible to their followers.
- “R”: the Recovered state. In Twitter it refers to the users that have retweeted the message, and their retweets have lost visibility to their followers.

The spreading starts with a single node in the state “I”, which is the source of the message. Then the follower nodes in state “S” each have probability *P*_*retweet*_ to retweet the message. Meanwhile, nodes in the “I” state can change to the “R” state, which means that their retweets become invisible to their followers because of the influx of other messages. The spreading process ends when there is no more “I” nodes; then the number of “R” nodes is the final popularity, referring to the total number of users that have retweeted the message (i.e. the number of retweets). For simplicity, the rest of our analysis assumes discrete time steps of spreading probability *P*_*retweet*_ over each link, and the recovery probability is 1 during each time step.

We use the self-consistent probability method on a directed network with in-degree and out-degree correlation to solve this problem. Since every retweeted user can be reached from the original user who created the message, we use the giant out-component to represent the cluster consisting of all retweeted users^39^.

6 $$\begin{array}{rcl}{q}_{out}({k}_{in}) & = & {P}_{retweet}({k}_{in})\sum _{{k}_{out}}\frac{{k}_{out}\times P({k}_{out})}{\langle {k}_{out}\rangle }\\  &  & \sum _{{k}_{in}}P({k}_{in}|{k}_{out})[1-{(1-{q}_{out}({k}_{in}))}^{{k}_{in}}],\end{array}$$

7 $${s}_{out}=\sum _{{k}_{in}}{s}_{out}({k}_{in})=\sum _{kin}\,P({k}_{in})[1-(1-{q}_{out}{({k}_{in})}^{{k}_{in}}].$$

Here *q*_*out*_ is the probability that the link following the inverse direction of information flow (inverse direction of the link) leads to a giant out-component and the receiver user (the end node of the directed link) that retweets the message. On the right side of the eq. (6), the second part is the probability that the out degree of the spreader user is *k*_*out*_; the third part is the probability that the spreader user has at least one incoming link that leads to the giant out-component and retweets the message over this link. If the first part *P*_*retweet*_(*k*_*in*_) is a constant, i.e. independent of *k*_*in*_, then the model becomes the standard SIR model in a directed network with in-degree and out-degree correlation. However, the realistic situation is complicated. Due to the competition for the limited attention and the heterogeneous retweet activities, the retweet probability is dependent on the in-degree of the receiver user.

From here, we can apply our empirical eqs (1–3 and 5) and solve the giant component size from the eqs 6 and 7, and compare with the results from the SIR model and empirical analysis.

#### Critical threshold for the information spreading

From eq. (6), we can calculate the giant out-component size (GC), which is the number of retweets from eq. (7).

**Figure 4:**
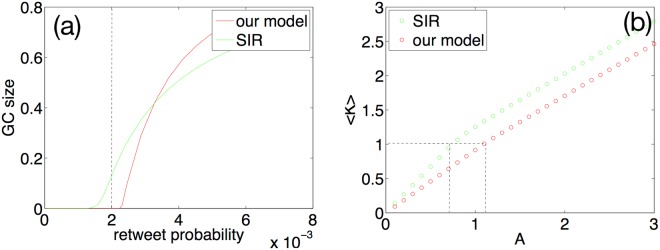
(**a**) The giant component size as a fraction of network size vs. the average retweet probability. The vertical line refers to the attractiveness value at 1.0. (**b**) The average out-degree of the retweet tree for various attractiveness values of the messages. The critical phase corresponds to 〈*K*〉 = 1. For our model, the critical threshold for attractiveness is larger than 1, in contrast to the value of smaller than 1 in the SIR model.

Since the number of Twitter users is approximated as a large graph, the phase transition happens when a nonzero fraction of the users retweets the message. In other words, the phase transition happens when *GC* > 0, which means that the message spreads out and has a relatively large retweet popularity. For different retweet probabilities, we can calculate the corresponding *GC* size. Figure 4(a) shows the analytical results for the giant component size over the retweet probability for our model and the SIR model. For comparison, the giant component size is plotted versus the average retweet probability over all links, represented as: $\langle {P}_{retweet}\rangle ={\sum }_{{k}_{in}}\,\frac{{k}_{in}P({k}_{in})}{\langle {k}_{in}\rangle }{P}_{retweet}({k}_{in})$. In Fig. 4(a), we can see that in the SIR model, messages can spread out (*GC* > 0) even with *A* < 1 (less than average attractiveness). This does not correspond to the empirical observations that only a small fraction of tweets spread out, and they are usually the ones with high attractiveness. Our model is able to demonstrate the pattern that only *A* > 1 messages can spread out.

**Figure 5:**
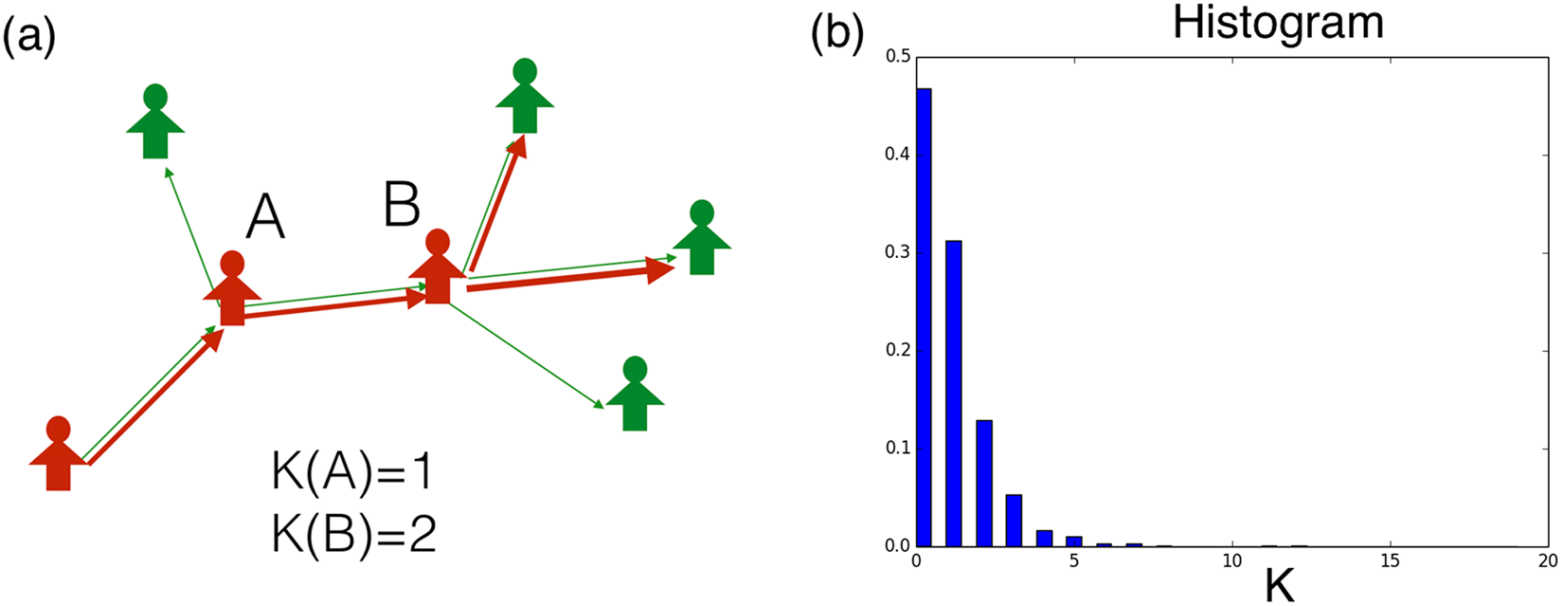
(**a**) Schematic diagram of the retweet tree. The out-degree of the retweet tree represents the number of followers that retweet the message from a specified node. (**b**) The distribution of the retweeting out-degree *K* for a viral tweet.

We can also investigate the critical threshold from another point of view. For convenience, we name the retweet chain composed by different generations of retweeted users as the retweet tree. As shown in Fig. 5(a), the retweet tree is illustrated by red nodes and edges.

For a branching process in the directed network with a known network structure, the average out-degree of the retweet tree can be calculated, represented as 〈*K*〉. It can be deduced from our model as

8 $$\langle K\rangle =\sum _{{k}_{out}}\,P({k}_{out})\times {k}_{out}\times \sum _{{k}_{in}}\,\frac{{k}_{in}\times P({k}_{in})}{\langle {k}_{in}\rangle }\times {P}_{retweet}({k}_{in}).$$

Since 〈*K*〉 depends on the retweet probability, which is tuned by the attractiveness *A*, Fig. 4(b) shows the average out-degrees of the retweet tree over the attractiveness of messages. For the branching process, the critical condition of the phase transition is 〈*K*〉 = 1^40^. Such a critical threshold corresponds to attractiveness *A* > 1 in our model, contrary to the SIR model. In other words, our model is more realistic than the SIR model since only messages with attractiveness higher than the average value can spread out virally.

#### In-degree histogram of retweeted users

In order to validate our model with the empirical data from Twitter, we deduced the in-degree distribution of the spreading tree from both the SIR model and our model, and then compared them with statistical results of empirical Twitter messages.

The in-degree distribution of the retweeted users should be the ratio of the number of retweeted users with a specific in-degree to the total retweet users as follows:

9 $${P}^{R}({k}_{in})=\frac{{s}_{out}({k}_{in})}{{s}_{out}}=\frac{P({k}_{in})[1-(1-{q}_{out}{({k}_{in})}^{{k}_{in}}]}{\sum _{{k}_{in}}\,P({k}_{in})[1-(1-{q}_{out}{({k}_{in})}^{{k}_{in}}]}.$$

**Figure 6:**
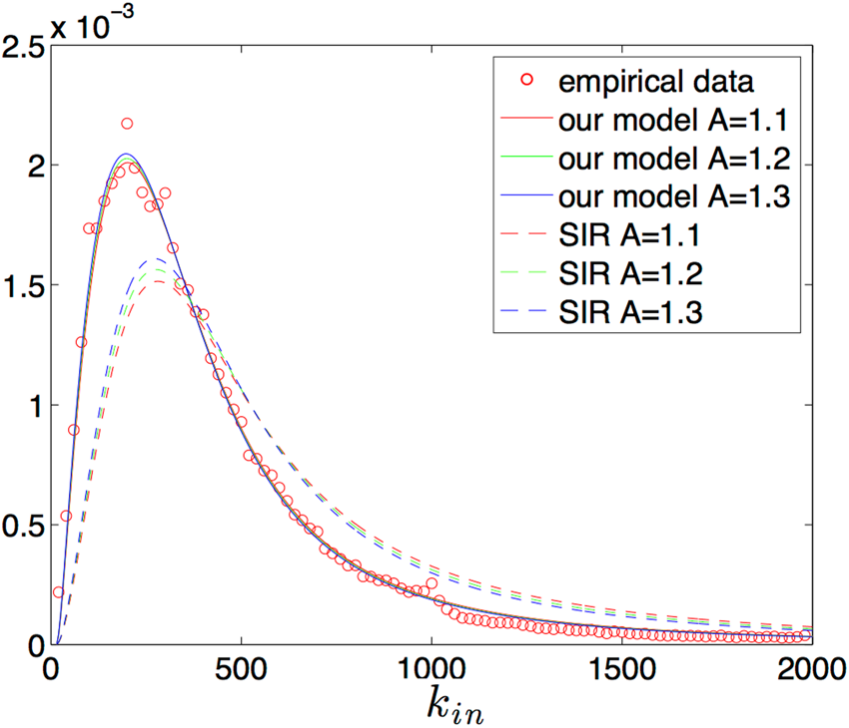
The PDF of in-degrees of retweeted users. The analytical lines with different colours show that the in-degree histograms for messages with various attractiveness (A = 1.1, A = 1.2 and A = 1.3) have slight differences. The dashed line is the result for the SIR model, the solid line for our model and the points from the empirical statistical result.

Here, *s*_*out*_(*k*_*in*_) represents the number of retweeted users with a specified in-degree value and *s*_*out*_ represents the total number of retweets. The analytical results of the above equation for both the SIR model and our model are compared with the empirical statistical results as shown in Fig. 6. Since we focus on the analysis of viral messages, their attractiveness should be above the average *A* = 1. The analytical results of our model with different attractiveness are close to the empirical observations, whereas those of the SIR model do not. This is further evidence that our model captures the spreading process better than the SIR model.

## Statistical Properties of Retweet Tree and Popularity Prediction

### Popularity and Statistical description of the out-degrees of the retweet tree

In this section, we investigate the properties of the retweet tree in relation to the retweet probability *P*_*retweet*_. From the empirical data of Twitter retweets, we construct the retweet tree by tracking the connections between the retweeting users as shown in Fig. 5(a). From eq. 8, we know that the out-degree of the retweet tree is closely related to the retweet probability. Hence, we can investigate the distribution of the out-degrees *K* of the retweet tree. The distribution of *K* is illustrated in Fig. 5(b) for a particular tweet.

**Figure 7:**
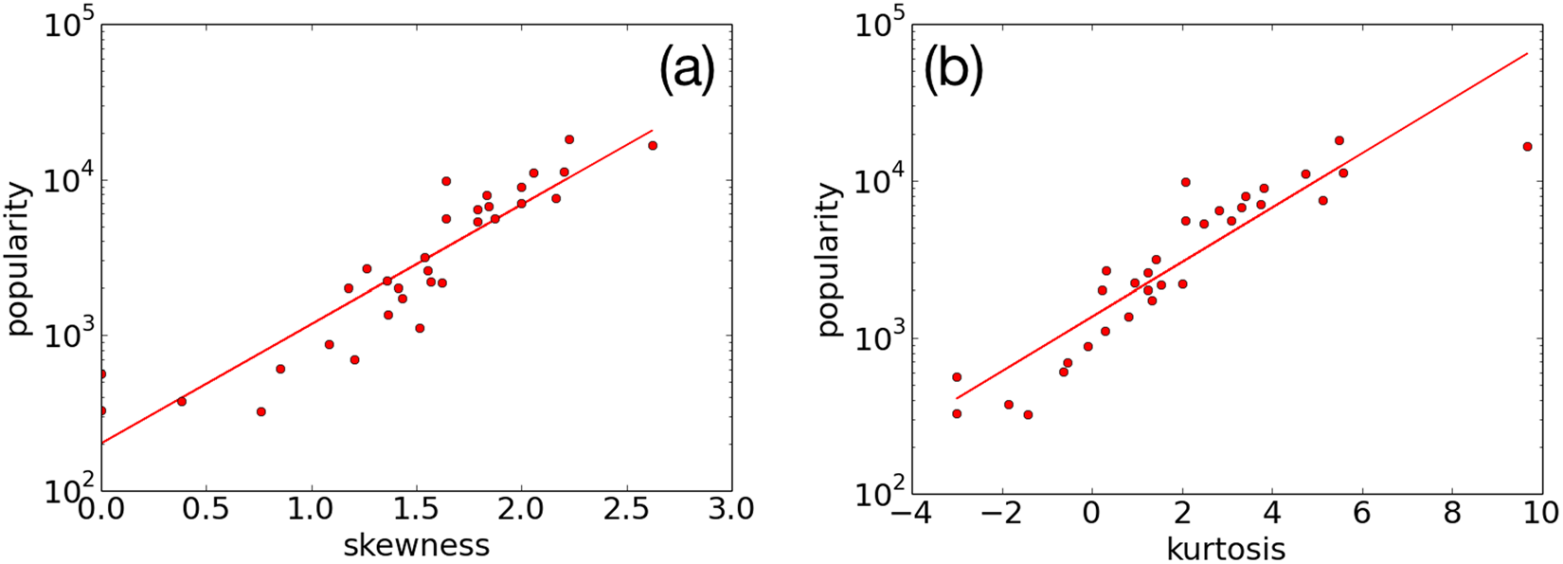
Statistical properties of the retweet tree nodes. Each point is a retweet tree for a viral tweet. (**a**) Skewness of the out-degree distribution of the retweet tree vs. the popularity of retweet. (**b**) Kurtosis of the out-degree distribution of the retweet tree vs. the popularity of the retweet. A clear positive correlation is observed.

We now explore the properties of kurtosis and skewness that measure the “tail” and asymmetry of the probability distribution of *K*. As shown in Fig. 7, the popularity is positively correlated to the kurtosis and skewness of the *K* distribution. Next we investigate on this relation in our model.

### Retweet branching process simulation

To get a theoretical understanding of the retweet tree, we use numerical simulation to calculate the retweet number in each time step as follows. A branching process starting with an initial node can be described by the following iteration equations. ${N}_{t}^{i}$ represents the number of new retweeted users in time step *t* and ${N}_{t}^{R}$ represents the total number of retweeted users at time step *t*. In the initial state, we set ${N}_{0}^{i}=1$, ${N}_{0}^{R}=0$, ${N}_{0}^{S}=N-1$. Then the branching process iterates as follows:

10 $${N}_{t}^{i}={N}_{t-1}^{i}\times \sum _{{k}_{out}}\,{P}^{R}({k}_{out})\times \langle {P}_{retweet}\rangle \times {k}_{out}\frac{N-{N}_{t}^{R}}{N}$$

11 $${N}_{t}^{R}=\sum _{t^{\prime} =0}^{t-1}\,{N}_{t^{\prime} }^{i}.$$

The term $\frac{N-{N}_{t}^{R}}{N}$ represents the fraction of the remaining susceptible nodes. *P*^*R*^(*k*_*out*_) is the out degree distribution of the retweeting nodes. It can be deduced from *P*^*R*^(*k*_*in*_) in the previous section shown in eq. (9):

12 $${P^{\prime} }^{R}({k}_{out})=\sum _{{k}_{kin}}\,{P}^{R}({k}_{in})\times P({k}_{out}|{k}_{in}).$$

**Figure 8:**
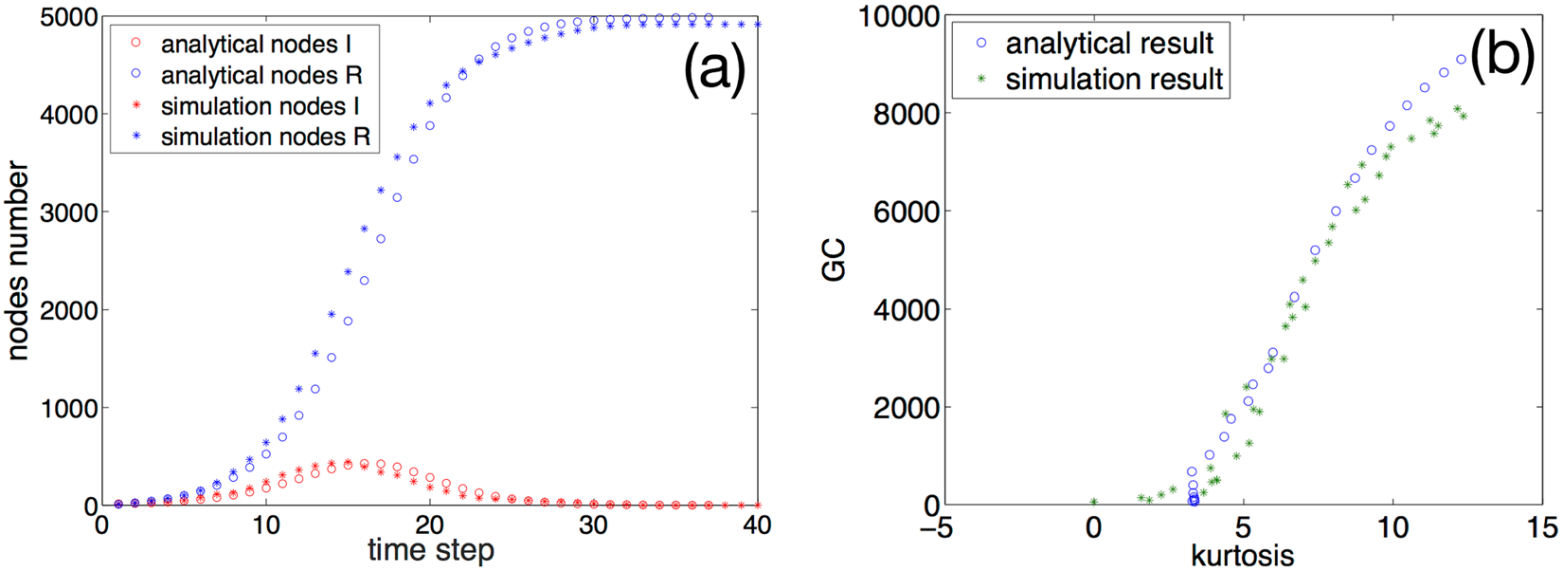
(**a**) Time evolution of the retweet process from our numerical method (points) and network simulation (star line). The results are close, validating our analytical framework. (**b**) The giant component size vs. kurtosis. The GC size increases as the kurtosis increases. The circles represent analytical results through the numerical method and the stars represent the simulation results of spreading in the generated network. The analytical and simulation results agree well.

Our numerical results on the evolution of ${N}_{t}^{i}$ and ${N}_{t}^{R}$ are compared against the spreading simulation on an artificial network with structural properties the same as the empirical Twitter network. As shown in Fig. 8(a), the two results are consistent with each other, demonstrating the validity of eq. 10. From here we analyze the relation between popularity and the statistical properties of the retweet tree. With the known *K* distribution, we can calculate the corresponding kurtosis of the *K* distribution both for the numerical result and the empirical results of Twitter messages. To compare the kurtosis of various messages, we change the retweet probability by tuning the variable attractiveness. Meanwhile, from section IIB, we can calculate the GC size (i.e. the total number of retweets) under different tweet attractiveness. In this way, we can establish the relationship between kurtosis of the out-degree distribution of the retweet tree and the GC size analytically through our method. The result is showed in Fig. 8(b). We can see that the GC size increases as the kurtosis increases, and the analytical results match closely with the simulation results. Such positive correlation is also reflected in the empirical data from Section IIIA.

### Popularity prediction using the out-degree distribution of retweet tree

Our theoretical and empirical analyses established a robust relationship between the popularity and the distribution of the out degree *K* of retweet tree. It inspires us to extend this relationship to a practical solution of popularity prediction of viral tweets in its early stage of spreading. Theoretically, the spreading never stops until a global infection results. In such a case, the 〈*K*〉 has a value larger than 1 and should have a positive correlation with the final popularity. However in real social networks, the spreading stops after a certain time period because of the attractiveness loss (for example, after a political election result is announced or a blockbuster is no longer in cinema). The stop of the spreading leads to large numbers of peripheral nodes (without followers retweeting, i.e. *K* = 0) so that the empirical 〈*K*〉 can be very close but smaller than 1. Hence, it is impractical to use 〈*K*〉 for popularity prediction. However, the kurtosis and skewness of the *K* distribution has positive correlation with final popularity which inspire us to exploit the full information of the *K* distribution for popularity prediction.

**Figure 9:**
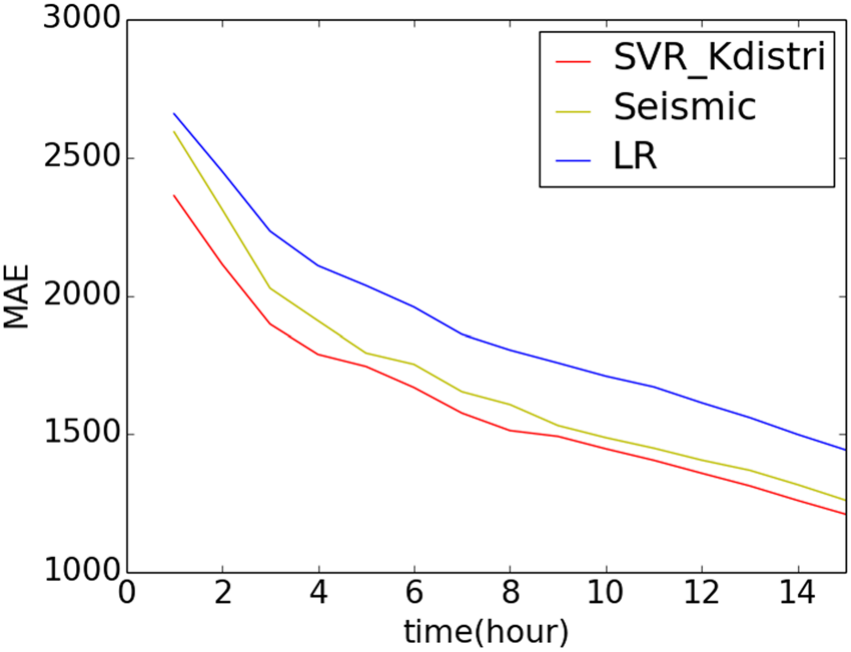
Evaluation of the model performances by mean absolute error (MAE) using our SVR method from Kdistri features (red line) compared with the linear model (blue line) and the SEISMIC model^42^ (yellow line) at different time t during the spreading process. Each model is calibrated at every hour after each tweets started spreading. Our model has the lowest error compared to the other two.

We use the support vector regression method (SVR) with linear kernel to predict the final popularity. For the input of the prediction model, we use the retweet number and out degree *K* distribution of the spreading tree at time *t* after the spreading started from the seed node. Since the tail of the *K* distribution does not have reliable statistics in the early stage due to limited sample size at the early stage of spreading, we divide the *K* distribution into four bins and obtain a vector of length four, with each element in the vector given by the proportion of *K* falling in each bin. Combined with the retweet number at time *t*, we construct an input vector of length five and use the SVR method to predict the final retweet number. The tweets in dataset is crawled from Twitter stream during the period of March, 2017 to Oct, 2017. In order to remove potential bot activities, we apply the entropy based classification method^41^ to filter out the tweets with relatively lower time interval entropy for the same user entropy. The filtered dataset has 466 tweets. We use 233 tweets as the training set and the rest as testing set. We compared the prediction result with two existing models - linear regression model and SEISMIC model^42^ - and the result is shown in Fig. 9. The linear regression model used popularities at different times and produced linear correlations between early retweet numbers and final popularities. The SEISMIC model proposed by Zhao^42^ builds on the theory of self-exciting point processes to develop a statistical model. The mean absolute error (MAE) between the prediction and the empirical retweet popularity is shown and our model (labelled as “SVR_Kdistri”) outperforms the other two models.

The main advantage of our model is using the information from retweet tree, which is inspired by our mechanistic model. Since the attractiveness of empirical messages cannot be reliably deduced directly because of the existence of peripheral nodes on the retweeting tree, we introduce the feature of degree distribution extracted from the retweet tree. Because the retweet probability affects the out degree of the retweet tree, the intrinsic attractiveness is embedded in the structure of the retweet tree, which helps to improve prediction performance.

## Conclusion

In this work, we analyze the empirical network structure and behavioural patterns of Twitter, and incorporate them into a mechanistic model of spreading. In particular, we include the empirical user retweet behaviour characteristic and attention competition related to the in-degree (their friends). An attractiveness parameter *A* is introduced to describe the quality of the message that influences the spreading rate. We find that such attention competition characteristic calibrated on empirical data leads to retweeting statistics that align with empirical findings, in contrast to the original SIR model of spreading.

From the extension simulation of the model, we find a robust relationship between the popularity of the message and the degree distribution of its retweet tree. Both our analytical and simulation results show positive correlation that is consistent with empirical analysis of Twitter messages.

Inspired by the correlation, we propose a new method to predict the final popularity using the out-degree distribution of the retweet tree during the early stage of spreading. Our model shows better performance than other existing methods because we use the degree structure of retweet tree that carries the information of message’s intrinsic attractiveness.

In conclusion, our study proposes a mechanistic model based on empirical data analysis and develops a new approach to the population prediction, based on the structure of the retweet tree. These results may have great potential in public opinion forecasting related to social media marketing.

## Data Source

The data analyzed in this paper is obtained through Twitter’s public API. We have used 466 tweets for data analysis with retweet number in a range from 200 to 20000. During the message retrieval, in order to filter out messages spreading in the subcritical case, we carried out a filtering process. The filter constraints are as follows: total retweet number larger than 200; the ratio between total retweet number and follower number of source user (the user who created the message) larger than 0.5. This would filter out those messages that spread mostly in the seed node’s nearest neighbours. With the filtering of tweets that meet our criteria, we found that the fraction of viral tweets according to our definition is about 1.5% of all tweets. The method on estimating this fraction is as following: The tweets collected through streaming are random retweets in real time. Since the probability of the tweets shown in the stream is biased – tweets with larger retweet numbers would have a larger probability of being shown in the stream – the raw fraction of the filtered tweets is not a true reflection of the real fraction of filtered tweets. Here we let *R*_*i*_ represent the retweet number of tweets *i*, *M* is the set of tweets that apparently meet our criteria and *N* is the total set of tweets that came to our stream. The fraction of messages that actually meets our criteria can be calculated as $P=\frac{{\sum }_{i\in M}\,\frac{1}{{R}_{i}}}{{\sum }_{j\in N}\,\frac{1}{{R}_{j}}}$, and is estimated to be 1.5%. We collected ids and friends lists of retweeted users for each message that meets our criteria. Each retweet contains the information about the retweeted user id and the retweet time. Since the information about former retweet user along the retweet tree is unavailable from the API, we built a hash table to store the friend list of the retweeted users. Through a search algorithm, the friends that had retweeted the message before the retweeted user can be obtained. Since the numbers of friends that retweet the message can be more than one, we regard the most recently retweeted friend as the former retweeted user. Eventually the whole retweet tree with the information of the retweet time can be obtained.
